# Play-Based Activities with a CoderBot Robot on a Pediatric Ward: A Case Study

**DOI:** 10.3390/healthcare10071209

**Published:** 2022-06-28

**Authors:** Marco Castiglioni, Cristina Liviana Caldiroli, Alessandro Antonietti

**Affiliations:** 1 Department of Human Sciences for Education “Riccardo Massa”, University of Milan-Bicocca, 20126 Milan, Italy; cristina.caldiroli@unimib.it; 2 Department of Psychology, Catholic University of Sacred Heart, 20123 Milan, Italy; alessandro.antonietti@unicatt.it

**Keywords:** robot-based activities, hospitalized children, psychological health, well-being, CoderBot, positive emotion, single-setting

## Abstract

Being hospitalized is a threatening and stressful experience for many children. From a psychological point of view, children may experience increased feelings of anxiety and fear that can negatively influence their behavioral, cognitive, and emotional outcomes. To mitigate such adverse effects on children’s mental health and well-being, interventions that might contribute to protecting the emotional domain of hospitalized children are welcome. The present case study of a single-setting intervention allowed us to evaluate the impact, on children admitted to a pediatric short-term recovery ward (*N* = 61), of participating in play-based activities with a CoderBot robot. The methodology spanned multiple data sources (children, parents, nurses), field observation, and a sequential (quantitative–qualitative) mixed-method approach to data analysis. We found that robot-based activities are associated with enhanced well-being (particularly positive emotions). Both the participating children and their caregivers reported that the activity was enjoyable and interesting, especially thanks to its technologically innovative nature. We critically discuss these positive findings in relation to the strengths of our pilot study, as well as its contextual and methodological limitations, and outline possible future lines of development for this kind of project.

## 1. Introduction

This paper focuses on the use of robots as tools for improving the well-being of hospitalized children admitted to pediatric wards. During their stay in the hospital, children may undergo medical procedures, causing them to experience pain and distress [1]. This can result in longer recovery times and more extended hospital stays, giving rise to prolonged suffering [2].

There is a vast and well-established body of literature on the hospitalization of children (especially prolonged hospitalization), including in relation to the psychological effects of hospitalization on the child, the relationships between families and hospital staff, “school in hospital,” clown therapy, and other play-based interventions designed to support hospitalized children’s psycho-physical well-being [3,4,5,6,7,8,9,10,11]. 

Although robotic technologies are increasingly being deployed in health care and hospital settings to serve functions ranging from surgery to rehabilitation to foster the well-being of patients and staff [12,13], studies examining the use of robotics on pediatric wards for play-educational purposes are still relatively scarce [14,15].

In an example of existing work in this field, Messias et al. [16,17] described a robotic platform designed to offer edutainment (the term *edutainment*—which combines the words “education” and “entertainment”—is a neologism coined to express the potential, and the need, for teaching and learning to take place while having fun.) on pediatric-oncology wards in Portugal as part of the MOnarCH project. However, to date, this interesting and innovative research project has primarily been focused on identifying the optimal technical characteristics of robots for use in hospital settings, which impose severe physical and social constraints. Assuming that recreational–educational robots play a positive role in the psycho-physical well-being of young patients, the authors’ discussion revolves around robotic-engineering programming and technical issues (i.e., robot movement capacity, level of autonomy, and interactivity), without directly addressing the salient psychological dimensions (cognitive, emotional, relational, social, etc.) of the hospitalized child. Thus, rigorous empirical evidence concerning the effectiveness of deploying robotics to enhance the mental well-being of young patients is still lacking. 

In other interesting projects, researchers in the Veneto region of Northern Italy paid greater attention to the psycho-social and emotional effects of playful interaction between children and robots. More specifically, Baroni and Nalin [18] first examined the critical factors affecting the hospitalized child that can negatively influence therapeutic efficacy and the patient–doctor relationship, going on to analyze possible applications of robotics as “companion therapy”, comparing it to pet therapy. For obvious hygienic and sanitary reasons, it is not feasible to bring animals onto a hospital ward. Hence the concept of introducing anthropomorphic robots, which—given their humanoid appearance and the fact that they behave like living entities—might, like animals in pet therapy, help to reduce anxiety and stress in hospitalized children, enhancing their response to treatment, sense of self-efficacy, and motivation to care. Relatedly, a study by Nalin and colleagues [19,20,21] on children’s expectations and representations surrounding humanoid robots showed that the child tends to attribute psychological characteristics—such as the ability to feel emotions, engage in pro-social behaviors, and have friends or family—to these machines.

In 2016, two further promising robotics projects were carried out in pediatric settings at Padua Hospital, under the supervision of the Department of Women’s and Children’s Health of the University of Padua. The Baby Goldrake project deployed the humanoid robot Nao, which had been programmed ad hoc to engage in play activities with hospitalized children on the pediatric surgery ward and in the hospital’s schoolrooms. The second project, conducted in collaboration with the Department of Information Engineering and Fisher Italy [22], used the same robot as a form of “non-pharmacological therapy”, with a view of managing anxiety in children undergoing invasive procedures at the hospital’s Department of Palliative Care and Pediatric Analgesic Therapy. The aim of “non-pharmacological therapies,” which include a wide range of interventions (cognitive, behavioral, physical, interactive…) [23,24,25], is to mitigate anxiety and fear and, consequently, to reduce the quantity of sedative medication that must be administered to children in preparation for painful and invasive examinations, such as biopsies and endoscopies, e.g., [26,27]. In the second Padua Hospital experiment, the robot Nao first welcomed the young patients into the hospital room, took them by the hand, and accompanied them to their bed; then, based on their requests, it entertained them by playing, singing, dancing, and telling them stories. 

These innovative projects are comparable to studies by Beran and colleagues with pediatric patients undergoing a variety of painful medical procedures, e.g., [28,29,30], and children with cancer [31]. The data collected in the above studies are encouraging, albeit that for the time being they concern a limited number of subjects and are thus not amenable to statistical generalization.

The projects just reviewed clearly reflect a growing interest in using robots to enhance the well-being of hospitalized children. The present study follows this line of inquiry. However, it is not based on a classical experimental design; Rather, it is more akin to the field observation of a single case. Its lack of a control group precludes systematic comparisons between the activities carried out with the robot and other recreational activities offered in the playroom of the ward. The onset of the COVID-19 pandemic, combined with the numerous constraints imposed by the hospital setting (see below), prevented the researchers from conducting other initially planned activities and from controlling for a set of potentially confounding factors.

## 2. Materials and Methods

### 2.1. Objectives of the Study

This exploratory field study may be viewed as a pilot project for informing future research. Its aim was to evaluate whether an activity performed with a mobile robot (designed for play and educational purposes) positively influenced the emotions of hospitalized children. Defining the term “emotion” points us to a vast range of theories and constructs in the literature, many of which suggest that emotion is actually multicomponent in nature. For the purposes of this study, we adopted the theoretical model of emotion that underpins the PANAS-C 30 (Positive Affect and Negative Affect Scales) [32], a set of 29 self-report items that children are asked to rate on a five-point Likert scale expressing the frequency with which they have experienced each of the described emotions. Specifically, the instrument we administered to our participants (see Section 2.4) was based on the PANAS-C short form.

The study drew on a mixed qualitative–quantitative design, which included the administration of Likert-scale questionnaires to all the children who participated in the activities with the robot and semi-structured interviews to a subset of parents and hospital caregivers (nurses and volunteers) working with the young patients. This approach allowed us to: (a) examine whether and how the play activities with the robot influenced the children’s emotions, as well as their general satisfaction with the robotics offering; (b) access the opinions and suggestions of parents and hospital caregivers in relation to the robotic activities conducted. 

The study was carried out prior to the COVID-19 pandemic and all further activities planned by the researchers based on the preliminary data remain on hold at this time. 

### 2.2. Sample

The sample comprised 61 children admitted to a hospital ward in the Milan district, a large, industrialized area of Northern Italy. The sample was gender-balanced (54.1% male) and the children were 9 years old on average (*M* = 105 months, *SD* = 36.4, range = 48–168 months). In terms of age, the difference between boys (*M* = 100.3; *SD* = 32.5) and girls (*M* = 110.6; *SD* = 40.55) was not statistically significant. The most frequent reasons for hospitalization were: respiratory disease (*N* = 9, 14.7%), unclassified pain (*N* = 8, 13.1%), orthopedic issues (*N* = 8, 13.1%), and pediatric appendicitis (*N =* 8, 13.1%). This was a convenience sample, for which hospitalization at the host facility and age (4–14 years) were the inclusion criteria. The patients on the ward ranged from infants to teenagers close to their eighteenth birthday, but we chose the—already wide—4–14 years age range, excluding both very young children and older adolescents, because including these further categories would have made the sample too heterogenous. Initially it had been planned to divide the sample into more homogeneous age bands (preschool: 4–6 year; primary school: 7–10 year; lower secondary school: 11–14 year) and to recruit a larger number of participants to each of these subgroups. However, the advent of the COVID-19 pandemic meant that this program could not be implemented.

Admission times at this facility were very short, averaging 2–3 days. Participants’ eligibility was verified by means of a medical history questionnaire completed by the children’s parents. The inclusion criteria were constrained by the characteristics of the pediatric ward, in particular the wide range of illnesses for which the children had been admitted and the fact that they were all short-stay patients. However, despite the diversity of their medical conditions, at the time of the study all participants were able to get out of bed and go to the room where the games with the robot took place. In addition, the fact that this was a short stay ward effectively excluded patients with chronic diseases or conditions requiring longer periods of hospitalization, such as cancer, chemotherapy, etc. 

The children and their families participated in the trial on a strictly voluntary basis. In agreement with hospital management, all parents of new patients received a description of the research project and a voluntary consent form together with the admission documents on arrival at the pediatric ward. Before participating in the study, an investigator individually briefed each child about the research, both verbally and via an information sheet that the participant was required to signed; The researchers gave the same information, both oral and written, to the parents, who provided written informed consent and authorization for the researchers to handle their children’s data as required under Italian data protection legislation.

### 2.3. Procedure

We obtained approval for the study from the human research ethics committee (Commissione Etica per la Ricerca in Psicologia CERPS, Department of Psychology, Catholic University of the Sacred Heart, Milan, Italy). On behalf of all the authors, the corresponding author states that there is no conflict of interest.

The study was conducted in a pediatric unit at a medium-size public hospital located near the city of Milan. The unit (15 hospital beds) was dedicated to young patients aged between 0 and 17 years. The pediatric unit also provided an emergency room service open 24 h a day. In addition to patient rooms, the pediatric unit comprised several other spaces, including a playroom. The doctors’ room was free in the afternoon and hence could be used as a dedicated space for the activity with the robot, given that no other extra space was available. Due to the size and internal layout of the room, only individual (and not group) activities were allowed. The activity took place on two to three afternoons a week, as allowed by department management, when the doctors’ room was not in use for meetings or other hospital activities. 

**Figure 1:**
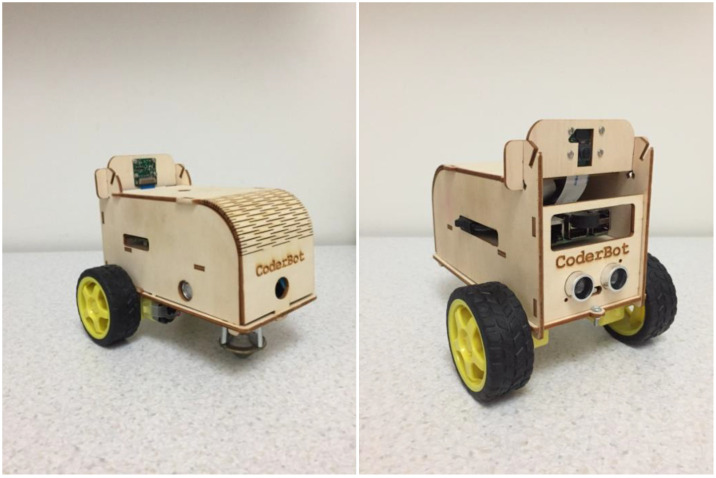
CoderBot Images.

The robot used was a type of “CoderBot” [33], an “open source” robot equipped with a camera and ultrasonic sensors, which could be remotely operated via a simple web interface and programmed using various graphical and textual languages. It was a small wooden vehicle with two independent front-drive wheels and a third support point at the back consisting of a freely rotating sphere (see Figure 1). It did not therefore present a humanoid or anthropomorphic appearance, nor that of a small animal, unlike the robots used in some of the studies described above. 

The robot was controlled via a PC interface. Participants could observe both the robot’s behavior on the floor and the image acquired by the robot’s front camera, which was reproduced in real-time on the PC screen. On the computer screen, next to the image transmitted by the robot, a set of commands were displayed—“forwards”, “backwards”, “right”, and “left”—which the participant could select using a mouse to make the robot perform the desired movements.

**Figure 2:**
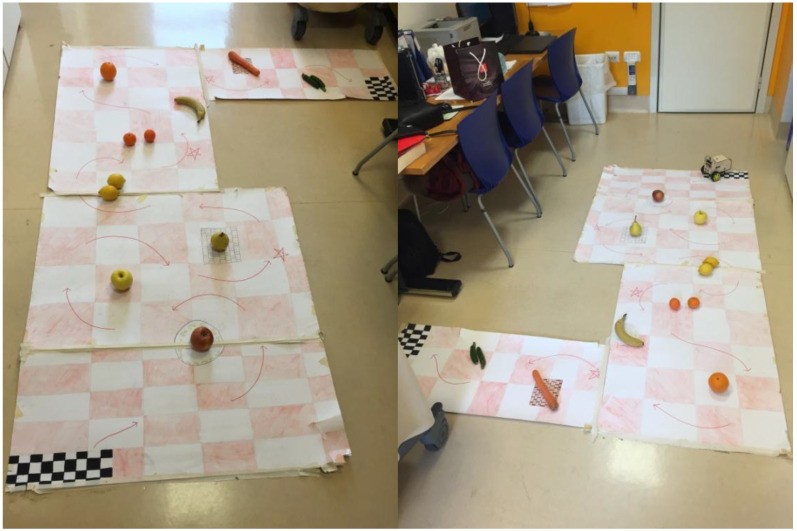
Example of complex obstacle course for 9- to 11-year-old children.

The proposed activity consisted of guiding the robot through an obstacle course. The trajectory was marked out on large sheets of card with a chessboard background, which were taped to one another and to the floor. Coloured arrows indicated the direction that the robot needed to follow to reach the flag marking the finish line. Pieces of fruit were positioned along the path to act as obstacles. The difficulty of the obstacle course increased as a function of age. Preschoolers were required to drive the robot about without allowing it to collide with any of three obstacles. Children between 6 and 8 years were required to navigate five obstacles, along a trajectory that they were invited to follow in the direction marked by a set of special arrows. Finally, older children (9–14 years of age) were asked to navigate a more challenging trajectory with 11 obstacles, which were positioned further apart at the beginning of the pathway and closer together towards the end (see Figure 2).

The children completed the activity individually (i.e., one child at a time, in the presence of a researcher as “facilitator”, and sometimes of a parent and, when possible, a representative of the Children in Hospital Association—in Italian: Associazione Bambini in Ospedale, ABIO—as observers) in the dedicated venue (the doctors’ room, as earlier stated). 

If the children got stuck while guiding the robot through the trajectory, the facilitator helped them by formulating questions designed to stimulate their thought processes, but without providing ready-made solutions, given that identifying solutions was the child’s task. Other adults present (e.g., parents, ABIO volunteers) were not allowed to intervene in these cases. 

Each activity session lasted about 20 min. On arriving in the doctors’ room, the children were invited to sit in front of the computer, where they could comfortably use the PC and view the obstacle course, which, on slightly turning their chair, was positioned parallel to them. First, the facilitator introduced the small robot to the children and explained clearly and in detail what the activity consisted of and what they were specifically required to do. Following this initial phase of familiarization with the rules of the game and with the commands for the robot, the children completed the first questionnaire concerning their emotional state prior to engaging in the robotics activity (see Section 2.4 below). Next, the activity proper could begin. Participants were given the opportunity to experiment with maneuvering the robot for a few minutes before tackling the obstacle course. During the activity, the facilitator carefully observed the child, noting any particular or abnormal behaviors and intervening as little as possible with a view to allow the children to concentrate and express themselves freely and to creating a relaxed atmosphere that did not evoke having to take or pass a test. Once the participants had successfully completed the activity, the facilitator administered two further questionnaires to them (see Section 2.4). The final phase of the activity consisted of obtaining the participants’ feedback via a short semi-structured interview. An ad hoc semi-structured interview was also administered to some of the parents and hospital caregivers who had been present at the session (see Section 2.4). Finally, the facilitator accompanied the child (and caregiver) back to their room.

### 2.4. Instruments: A “Mixed Method” Data Collection Strategy

A case study generally involves the investigation of small, ethnographically grounded samples via participant–observation conducted on-site with a view to identifying specific properties of a single case or phenomenon and the collection of data from multiple qualitative and quantitative sources [34,35,36,37]. Hence, a *mixed-method* research design is deployed. The qualitative paradigm provides the general framework within which locally collected quantitative information (biographical data, observation grids, inventories, and questionnaires) may be situated. An excessive focus on the quantitative dimension or measurement can lead researchers to overlook the “interactive” dynamics between the case and its surroundings, thus failing to grasp the role of contextual features and constraints [38]. Following the tradition of program evaluation case studies [39,40], in the current study we set out to explore: (a) what happened during the intervention, (b) what kind of impact it had on the children’s emotional states, and (c) how the intervention was delivered and assessed by all the actors involved (children, parents, and nurses/volunteers). We gathered a combination of quantitative and qualitative information with a view of more fully accessing the complexity of the phenomenon under study. 

The participants completed two scales selected to quantitatively monitor the efficacy of the intervention: one for detecting any changes in the children’s emotional states (B1) and one for assessing the children’s satisfaction with the activity (B2). 

The first battery of questions (B1) examined five bipolar dimensions (happy–sad, strong–weak, interested–bored, calm–agitated, and quiet–scared) [32], with a view of evaluating the child’s emotional state before (t0) and after (t1) taking part in the activity. The instrument was based on a semantic differential technique [41], whereby respondents were asked to rate on a Likert scale their closeness/distance to each of two semantic poles (i.e., “happy vs. sad” or “useful vs. useless,” etc.). The overall reliability of the scale was good (α = 0.823). This instrument was administered both immediately prior to the activity with the robot (pre-test) and immediately after it (post-test), with a view of measuring any differences between the two timepoints regarding the child’s emotional state. Because participants’ hospital stays were brief, and due to constraints imposed by hospital management, it was not possible to conduct further follow up testing at a later timepoint. 

The second battery of questions (B2) assessed six bipolar dimensions (easy/difficult, useful/not useful, good/bad, fun/boring, pleasant/unpleasant, new/old) in relation to participants’ reactions to the robotics activity that they had just taken part in. This instrument was only administered once, at the end of the hospital-based intervention. The reliability coefficient for B2 was 0.91. 

**Figure 3:**
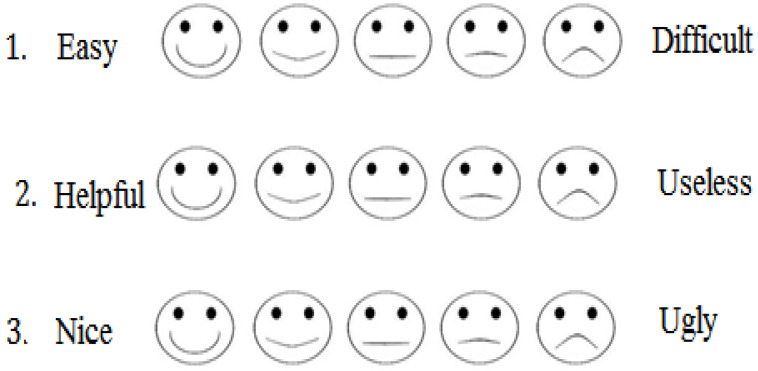
Examples of the modified (“smiley face”) Likert scales used with children under six years.

Children over six years of age responded using traditional Likert scales (ordinal scale with numerical values), a technique that has already been proven reliable for this age group [42]. In contrast, participants under six years of age responded using a visual analogue (see Figure 3). Subsequently, we standardized both sets of scores using conventional data normalization practices, see [43].

Following standard data analysis practices in the social sciences [44], we calculated the skewness and kurtosis values for each of the study variables in order to evaluate the distribution of the data sets and identify potential violations of normality assumptions. We also assessed the data with a view to identifying uni- and multivariate outliers; The criterion used was Mahalanobi’s distance [45] with a *p*-value of <0.001. Accordingly, four cases (two children who had been admitted to have their appendix removed and two to have their tonsils removed), all younger than 6 years, were omitted from the analyses. To test whether the changes in emotional state (B1) scores (Δt0 − t1) were statistically significant, thus supporting the hypothesis that the robotics intervention would be effective in enhancing emotional states, we conducted repeated measures tests [46]. This enabled us to calculate the size of any changes in mean scores, their level of statistical significance [47], and the relative effect sizes [48]. We followed two trajectories to evaluate the impact of age on emotional state and reactions to the intervention, respectively. To assess changes in emotional states (B1), we applied a generalized within-subjects linear model for repeated measures, setting it to covary with the age variable. In relation to the participants’ own evaluation of the intervention (B2), we conducted zero-order correlational analyses. To compensate for the risk of type I errors, we drew on the Bonferroni correction method with statistical significance appropriately set at *p* = 0.025 [49]. Concerning the power of the effects [50], we considered that values greater than 0.50 indicated a “large” effect, values greater than 0.30 a “medium” effect, values greater than 0.10 a “small” effect, and values less than 0.10 a “negligible” effect. 

With regard to the qualitative instruments, a set of semi-structured interviews were conducted with children, parents, and nurses to validate and integrate the quantitative data. The areas explored with the children included: (a) their previous knowledge of robots; (b) the robotics activity they had just performed; (c) their hospitalization experience with specific reference to play activities. Parents responded to three open-ended questions concerning the following: the strengths and weaknesses of the robotics activity just conducted; any changes in the child’s mood before, during, and after the activity; other possible play activities (robots excluded) that they would recommend for entertaining children in the hospital setting. Finally, nurses/volunteers answered four open-ended questions: (1) What is your overall impression of the robotics activity?; (2) What are the strengths and weaknesses of the activity in your opinion?; (3) Do you see any differences between the robotics activity and the “traditional” activities carried out in the playroom?; (4) Do you have any advice, or would you like to add something? Did you receive any feedback from any child who participated in the activity? Overall, we interviewed four nurses/volunteers and 18 parents of the hospitalized children that took part in the activities with the CoderBot. We fully transcribed the interviews and integrated them with the quantitative measures. We then analyzed the material collected from the children, their parents, and their hospital caregivers via a thematic analysis conducted by two independent judges.

## 3. Results

### 3.1. Quantitative Measures of Emotional States and Evaluation of the Activity

The main descriptive statistics for all (B1) measures of changes in emotional states are summarized in Table 1. With regard to the baseline measures, fearlessness (*M* = 4.02; *SD* = 1.29) and interest (*M* = 3.78; *SD* = 1.42) were the emotions most strongly felt by participants before beginning the robotics activity.

Interestingly, the set of emotional scores (B1) changed after the children participated in the robot-based activity. At t1, scores on three of the five emotions displayed statistically significant differences. The multivariate analysis of covariance revealed that, regardless of the children’s gender and age, they reported being significantly “happier” (F (1, 58) = 5.45, *p* = 0.038, η2 = 0.072) and “stronger” (F (1, 58) = 6.47, *p* = 0.014, η2 = 0.111) after engaging in the robotics activity. The effect size, in this case, was 0.11 (low). With regard to being “interested”, the pre-post change was not statistically significant. In contrast, a statistically significant effect of gender was found, i.e., boys (*M* = 4.64), more so than girls (*M* = 4.04), tended to report higher levels of interest following the activity with CoderBot. No significant differences were identified in relation to the other emotional dimensions. 

The outcomes of the evaluation of the robotics activity are summarized in Table 2.

Regarding the participants’ evaluation of the activity (B2), overall, the children rated the activity positively. In relation to which age group was optimal and reported the greatest effectiveness of the activity, a threshold effect occurred at about 120 months of age. Indeed, the scores of participants over 10 years of age did not change significantly following the intervention. Multivariate analysis of the cohort aged between 48 and 120 months (corresponding to the 4–10 year age group) identified a significant difference between mean scores at t0 and t1 (t (28) = 25.01, *p* < 0.001; η2 = 0.390) with an effect size of 0.40 (medium–high).

### 3.2. Qualitative Evaluation: The Participants’ Voices

We conducted thematic analysis of the textual corpus produced by the parents and hospital caregivers using a bottom-up approach [51]. The main recurrent themes are summarized below. The perceived strengths of robotics included its novelty value, cutting-edge profile, and technological appeal. These factors made the use of robots particularly attractive, especially for children in the intermediate age range (6–10 years), with the result that they remained interested and attentive for the entire duration of the activity. The younger children encountered greater difficulties, particularly in relation to using the mouse, which is still quite challenging for them at this age. Specifically, the difference between touch screens and mouse-controlled PC screens proved to be a hindrance. Children are more accustomed to using touch screen devices (parents’ smartphones, video games, etc.). It follows that implementing a “touch model” would make interaction with the robot more user friendly. For example, one 4-year-old girl, having tried unsuccessfully to play by pressing on the PC screen, began with frustration and annoyance to wave the mouse around in the air, in an attempt to make the robot work. The facilitator was obliged to intervene to help her resolve the issue. Another factor viewed as crucial by the hospital caregivers concerned the lack of opportunity offered by the tested activity format to use social skills in conjunction with the experience of operating the robot. Socialization and sharing with other peers are viewed as crucial to hospitalized children’s well-being. The individual nature of the activity imposed by the constraints of the setting meant that it made no contribution on this front, providing opportunities neither for cooperation nor for competition among peers. The ideas proposed for future robotics projects in pediatric settings included the organization of team games, designed to stimulate both these components of socializing. The spaces dedicated to the activity were viewed as adequate for purpose. However, our informants suggested that it would be more desirable to have access to ad hoc spaces (rather than a “repurposed” doctors’ room), especially with a view to organizing robotics games for groups, as recommended by the interviewees.

From our interviews with parents, it emerged that the robotics activity was appreciated and viewed as appropriate and enjoyable in the hospital context.

*“And yes, he liked it, before he was bored and he even said it and sad, and then he is hungry. He hasn’t eaten for three days, so he is hungry, and he found it entertaining, so it’s interesting, he liked it”*.

(mother, 42 years old)

The main strengths that the parents associated with the recreational use of robots included the fun nature of the activity and the value of engaging in it during a hospital stay. The parents’ overall impression of the activity was positive. Some believed that it should be a standard offering on pediatric wards, given its potential to capture the interest of children and young people, who, in the hospital setting, often do not feel like doing anything and lose their desire to play.

Robotics can stimulate children’s curiosity and keep them focused and attentive for the duration of the proposed activity. Some parents reported that their child often displayed no interest in going to the playroom or only stayed there for a very limited time; They were therefore surprised by the concentration and commitment that the child had devoted to playing with the robot.

*“I tried to take him to the playroom this morning, but he wanted to do everything and play with everything; He brought out the dinosaurs and played for a second but wanted to change [game] right away; In fact I didn’t think he would get through the entire activity here [the CoderBot obstacle course] and [I thought] that halfway through he would tell you to stop, but instead he was very interested”*.

(mother, 32 years old)

The robot appeared to hold particular appeal for children and young people, who are drawn to new technologies, which stimulate them to get involved and try out new experiences.

One limitation flagged by a parent concerned the duration of the activity, which was viewed as too brief and infrequent, as reflected in the following quote. Given that her son enjoyed the game with the robot, the mother would have preferred it to last longer.

*“So, the limitation is that it was relatively short for my son; It was fun. If it had lasted longer, maybe it would have been more interesting, more enjoyable for him; However, I realize that doing it in an office, maybe the department here is small, but if there were twenty children who needed to do it I realize that the time... and then definitely it’s interesting because if you are studying this kind of activity, it will definitely yield results, I hope satisfactory, for your sake”*.

(mother, 43 years old)

The interview data suggest that all the parents noticed a positive change in their child’s emotional state during the activity with the robot. Many stated that they were surprised by their children’s enthusiasm during the activity compared to beforehand, when they were bored and disinterested. The novelty factor was experienced as positive and the parents reported that their children seemed happy and serene during the activity.

*“He was very interested; novelty is always welcome; it’s hard for him to get excited about anything”*.

(mother, 32 years old)

### 3.3. Other Observations from the Field and Practical Issues

Field observations showed that when the researcher went into the patients’ rooms to invite them to take part in the research, many of them were visibly bored and disinterested.

This discontent increased in direct proportion to the age of the patients, who often spent most of their time in their room playing with their cell phones or tablets. At this juncture, the encouragement of the parents, who immediately saw this unexpected proposition as a positive stimulus and a way to constructively occupy their children’s time, proved valuable and decisive. If nurses were also present, they too would stimulate the patient to participate.

Generally, during the activity, the participants were silent and focused on what they were doing. Questions and requests for help were reduced to the minimum necessary. In some cases, the children only asked for more detailed information out of curiosity when they had completed the obstacle course. Some of the older children also questioned the researcher about the study itself, with a view to learning about the broader research context. 

The participants were calm and at ease with using the computer but were surprised that it was necessary to use the PC to control the robot. They enjoyed watching the robot move but became so engaged in this and in monitoring the robot’s trajectory that they often kept the same command arrow pressed for too long. The challenge lay in coordinating the robot’s movements while shifting their gaze from the obstacle course to the PC and vice versa. Only the youngest children—4/5 years old—were visibly lost and unable to use the tool; During the activity with the smaller patients, the facilitator intervened several times to help them position the cursor where they wanted it to go.

Some of the parents of children in this age group reported at the end of the activity that it was the first time their child had ever used a computer:

*“The computer is definitely a plus, being able to work it. Maybe it’s a little too difficult for a 4-year-old because he had never used one before.”*.

(mother, 31)

Contrary to the participants’ concerns prior to beginning the robotics activity, no difficulties arose due to medical/health aids (IVs, bandages, stitches, etc.) acting as an impediment.

## 4. Discussion and Conclusions

Playing with the CoderBot positively influenced, at least in the short term, the well-being of the participants in this study, especially in terms of enhancing their emotional state. Primarily, the participating children reported feeling happier, stronger, and more interested after the activity than they did before it. They also expressed satisfaction with the activity, especially in relation to its enjoyableness. The dimension of interest was a recurrent theme in both the participants’ questionnaire responses and the interviews conducted with parents and hospital caregivers. The activity was “interesting” above all because it is “new”, technological, and therefore also “of value”, insofar as it allows children (especially boys up to 10 years of age) to experience (and experiment with) sophisticated and technologically advanced devices. Yet these outcomes, however encouraging, should be treated with caution. 

A key problem associated with case study methodologies concerns the robustness of the conclusions. The data is restricted to a single set of observations, limiting the scope for transferring the results to other settings without losing ecological validity. Because in this study we only drew on internal triangulation of data (with-in case method) [35,52], we must be careful about generalizing our outcomes to other contexts. While it is important to select case characteristics for typicality, it is equally important to reflect on situational uniqueness, especially in relation to the complex interactions of the case with background/contextual conditions [53].

In this regard, the second class of limitations of this work is linked to the constraints imposed by the setting, in terms of the brevity of the children’s hospitalizations, the heterogeneity of their medical conditions, and the impossibility to engage them in “social” games. A first methodological fallout of these constraints concerns the very close temporal distance between pre-test and post-test, which suggests that follow-up research on the effectiveness of the intervention may be appropriate. 

The influence of the different medical conditions present in our sample could be even more important. The type, severity, and duration of the illness (and the associated degree of suffering), as well as the kind of therapies administered, can significantly affect the general conditions and, especially, the emotional resources of sick children. Not surprisingly, numerous studies have been conducted with long-term patients affected, for example, by leukemia or cancer and/or undergoing chemotherapy [14,15]. 

It is equally true that, in a context of exclusively short-term admissions, none of the young patients in this study were in clinically serious conditions; Furthermore, children who were so ill that they could not get out of bed were automatically excluded from the activity. However, it was not possible for the researchers to systematically monitor either the severity of the participants’ illnesses nor the level of invasiveness of their treatment regimes. The wide age range of the patients should also be noted. In this regard, Baroni and Nail [16] suggested developing differential activities and deploying different types of robot as a function of the type of illness and treatment. 

In essence, the exploratory nature of our field research and the consequent constraints posed by the specific context prevented us from conducting a systematic evaluation of the effects of playing with the robot or from separating them out from other potentially influential factors, such as participants’ gender, prior familiarity with robots, the presence or absence of a parent during the activity, etc. A final major limitation is the lack of an adequate control group to compare to the “experimental” group, precluding systematic comparison with the “usual” activities conducted in the playroom on the ward. In this regard, only a few—albeit interesting—insights emerged from the interview data. 

In conclusion, the future development of this project should ideally include the following aspects. First of all, a systematic study of the effects that playing with a robot exerts on young patients should be conducted, in relation to the type of medical condition from which they suffer, the recruitment of a larger sample, with more homogeneous age cohorts (preferably concentrated around primary school age) that are statistically comparable, and the monitoring of the origin and socio-cultural level of children’s families, including with a view of assessing the possible role of intercultural differences; in all of this cases, comparison with a matched control group will be required. 

This is clearly a highly ambitious goal that poses key methodological challenges in ecological settings. In addition, as far as the game with the robot is concerned, new activities that are more attractive to older children and easier to manage for younger ones need to be designed. These activities must be suitable for groups and spread over several sessions (i.e., not “one shot” only, but multiple encounters forming a structured trajectory). Lund and Nielsen [54] pointed out that younger children are generally more sensitive to “game interaction”, i.e., to interaction with the robot, while as children grow older, the objectives and the “technical” aspects of the game become more important to them. Furthermore, evaluation of the “emotional effects” over the medium-term (follow up) and of potential “cognitive” learning (through comparison with other educational robotics research, e.g., in the domain of the resolution of “errors”) is required; Again, this line of inquiry should draw on a mixed-method approach (qualitative–quantitative). Finally, in-depth interdisciplinary theoretical reflection (spanning the epistemological, psychological, and technical–robotic domains) and empirical experimentation in the field must be conducted to compare the use and effects of CoderBots with the use and impact of humanoid robots: An enterprise that will likely engage researchers for a long time to come.

Our research group was about to initiate follow up research to this pilot study when the COVID-19 pandemic broke out, preventing us from carrying out further work in hospital settings. At the time of writing, we cannot foresee if and when these activities may be resumed. 

## Figures and Tables

**Table 1:** Descriptive statistics for emotional states (B1) at baseline (t0) and post-activity (t1) for the entire sample (*N* = 61).

	Baseline (t0)		Post-Activity (t1)		Δt0 − t1
	M *	*SD*	M *	*SD*	
Happy–Sad	3.52	1.30	4.07	1.48	0.54
Strong–Weak	3.15	1.29	3.71	1.25	0.57
Interested–Bored	3.78	1.42	4.37	1.07	0.59
Calm–Stressed	3.77	1.39	3.94	1.44	0.17
Fearless–Scared	4.02	1.29	4.43	1.13	0.41

* Note: the range of mean scores = 1–5.

**Table 2:** Evaluation of the activity (B2): descriptive statistics (*N* = 61).

	Post-Activity Evaluation	
Items	*M* *	*SD*
Easy/Difficult	4.07	1.48
Helpful/Useless	3.71	1.25
Good/Bad	4.37	1.07
Fun/Boring	3.94	1.44
Pleasant/Unpleasant	4.43	1.13

* Note: the range of mean scores = 1–5.

## Data Availability

The data presented in this study are available upon request to the corresponding author.
